# Scaffolds Mimicking the Tumor Microenvironment for In Vitro Malignancy Models

**DOI:** 10.3390/biomimetics10100695

**Published:** 2025-10-14

**Authors:** Elisabetta Rosellini, Maria Grazia Cascone

**Affiliations:** Department of Civil and Industrial Engineering, University of Pisa, Largo Lucio Lazzarino 1, 56122 Pisa, Italy

**Keywords:** tumor microenvironment, 3D in vitro tumor models, biomimetic scaffolds, decellularized extracellular matrix

## Abstract

The tumor microenvironment (TME) plays a crucial role in regulating cancer cell proliferation, invasion, and drug resistance. Traditional two-dimensional (2D) in vitro models and animal models often fail to replicate the biochemical and biophysical complexity of human tumors, leading to low predictive power in preclinical drug screening. In recent years, scaffold-based three-dimensional (3D) in vitro models have emerged as promising alternatives, offering a more physiologically relevant context for studying tumor behavior. Among these, biomimetic scaffolds capable of replicating the composition, stiffness, porosity, and signaling features of the tumor extracellular matrix (ECM) are of particular interest. This review provides a comprehensive overview of scaffold-based approaches for mimicking the TME in vitro. After outlining the key characteristics of the tumor ECM, we discuss various scaffold typologies, including those based on natural, synthetic, and hybrid biomaterials, as well as decellularized ECM. Recent advancements in fabrication technologies, such as electrospinning and 3D bioprinting, are also highlighted for their role in replicating the geometric and mechanical features of tumor tissues. Special attention is given to the integration of vascular components and stromal cells to recapitulate the complexity of the TME. Finally, we explore current limitations and future directions, emphasizing the need for standardized and reproducible models, particularly in the context of personalized cancer therapy.

## 1. Introduction

Tumors consist of the uncontrolled proliferation of cells whose phenotype has been altered by one or more genetic mutations. If these clusters of altered cells leave their primary site of proliferation, they can migrate and penetrate blood vessels. From there, they can reach secondary sites, leading to metastasis, i.e., secondary tumors distant from the primary tumor.

According to the World Health Organization (WHO), cancer is the second leading cause of death worldwide, with one in six deaths globally attributed to cancer [1]. According to the GLOBOCAN 2022 report by the International Agency for Research on Cancer (IARC), officially released on 1 February 2024, the most recent year with complete and validated data is 2022, which recorded approximately 20 million new cancer cases and 9.7 million cancer-related deaths worldwide in that year [2,3]. The five most frequently diagnosed cancers in 2022 were lung (2.48 million cases, 12.4%), female breast (2.30 million, 11.6%), colorectal (1.93 million, 9.6%), prostate (1.47 million, 7.3%), and stomach (970,000, 4.9%) [2,3]. Lung cancer was also the leading cause of cancer death, responsible for approximately 1.8 million deaths (18.7%), followed by colorectal (900,000; 9.3%), liver (760,000; 7.8%), breast (670,000; 6.9%), and stomach cancers (660,000; 6.8%) [2,3,4].

While complete global data for 2023 are not yet available, preliminary estimates and modeling based on GLOBOCAN and other surveillance systems suggest that cancer incidence and mortality in 2024 may slightly exceed 2022 levels, with projections of approximately 19.3 million new cancer cases (excluding non-melanoma skin cancers) and around 10 million cancer deaths projected for 2024 [5]. These projections are driven by ongoing trends in population aging, demographic growth, and the persistent prevalence of major lifestyle and environmental risk factors, such as tobacco use, alcohol consumption, and obesity [5,6].

Long-term IARC projections indicate that by 2050, the global number of new cancer cases could exceed 35 million, marking a +77% increase compared to 2022, with the most significant growth expected in low- and middle-income countries due to reduced access to preventive and early-detection services [2,5,6].

Despite the constant increase in cancer incidence, oncological research has led to the development of screening techniques and therapies capable of reducing the mortality rate associated with these diseases, significantly increasing the life expectancy of cancer patients. According to the American Cancer Society (ACS), advancements in cancer research have prevented nearly 4 million deaths between 1991 and 2021 [3].

The development of anticancer drugs and therapies requires preclinical experimentation to certify their efficacy before proceeding to clinical trials. Currently, the gold standard method for this evaluation is two-dimensional (2D) in vitro models: human tumor cell lines of interest are cultured on a culture plate until they form a monolayer. The drug is then added to this monolayer, and cell viability is measured using spectrophotometric techniques, such as vital dyes, and the quantification of biomarkers associated with cellular functionality.

However, it has been observed that at least 95% of drugs that show promise in 2D models prove ineffective in clinical trials [4]. This significant discrepancy between in vitro results and clinical experimentation is due to the fact that fully developed tumor tissue cells are organized into a characteristic three-dimensional (3D) structure, which determines a set of physicochemical properties that inevitably influence the efficacy of the tested drug. 2D models fail to replicate this characteristic structure, thereby explaining their evident methodological limitations. Nevertheless, it should be acknowledged that 2D models remain indispensable for high-throughput screening of thousands of molecules across cancer cell lines, prior to in vivo validation.

Another widely used tumor model is the animal model. In this approach, human tumor cells are implanted into immunocompromised rodents, typically mice, where they are allowed to proliferate for several weeks. Tumor cells can grow and differentiate efficiently, as murine models lack immune defense mechanisms to counteract the proliferation of the xenogeneic tumor. However, the absence of an immune component and the intrinsic differences between the human and murine immune systems—such as the lack of interleukin-8 in mice—make the animal model incapable of accurately replicating the human immune response to tumors. Furthermore, in some models, such as ovarian cancer, the anatomical differences in the target tissue or organ between animals and humans lead to histological differences, which may explain why only 27% of drugs effective in animal models also prove effective in Phase 2 clinical trials [5]. Additionally, the inevitable euthanasia of laboratory animals has led the scientific community to question the ethical implications of this experimental methodology. At the same time, animal models remain more sophisticated than in vitro engineered models, as they contain diverse immune subsets, stromal components, and vasculature that partially reproduce human tumor physiology.

Therefore, in recent years, there has been a growing need to transition to an alternative modeling methodology, capable of more accurately replicating a fully differentiated 3D tumor tissue. The introduction of this new modeling approach, driven by the need to study tumor development and proliferation dynamics, has proven to be more effective in preclinical drug testing as well. This is because it better reproduces the defense mechanisms of tumor masses, compared to 2D or animal models. Among 3D models, those based on biomimetic scaffold structures, which can faithfully replicate the tumor microenvironment, are of particular interest. This is because it is well recognized that the growth of a tumor mass, like any other tissue, is strongly influenced by the surrounding microenvironment, particularly the composition and mechanical properties of the extracellular matrix (ECM) [5,6,7,8,9].

The objective of this review is to investigate the role of scaffolds mimicking the tumor microenvironment in the development of predictive in vitro tumor models. After discussing the main characteristics of the tumor ECM and how this influences tumor tissue proliferation, scaffolds and their ideal properties for this type of modeling will be introduced. Subsequently, the biomaterials and fabrication techniques used will be examined, referencing key studies on in vitro tumor models. Finally, the advantages and prospects for improvement of these models will be discussed, with a specific focus on the scaffolds employed.

## 2. The Tumor Microenvironment

All human body cells are embedded within a specialized microenvironment that regulates their growth, differentiation, and communication with surrounding tissues. Beyond providing structural support, the ECM orchestrates a dynamic biochemical and mechanical signaling landscape. This matrix—comprising collagen (primarily types I and III), elastin, fibronectin, laminin, proteoglycans, and glycosaminoglycans such as hyaluronic acid—acts as both a scaffold and a reservoir for growth factors and signaling molecules [10,11,12]. In addition to ECM components, stromal cells (notably fibroblasts) deposit collagen and maintain matrix homeostasis, while immune cells such as macrophages modulate inflammation and tissue remodeling [13]. Enzymes including matrix metalloproteinases (MMPs) facilitate ECM degradation and turnover, and vascular structures deliver nutrients and oxygen while imposing mechanical forces, such as pressure gradients and shear stress, that profoundly influence cell behavior [11].

Upon malignant transformation, these tightly regulated processes become dysregulated. Tumor cells actively remodel their surrounding environment by increasing MMP expression, degrading basement membranes, and reshaping tissue architecture, thereby establishing the tumor microenvironment (TME), which diverges markedly from its healthy counterpart [13,14]. While tumor heterogeneity across organs, invasion patterns, and morphologies is well documented, recurring and defining features of the TME have emerged. These features, spanning both chemical and mechanical dimensions, support tumor growth, immune evasion, and metastatic dissemination [14,15].

One hallmark of the TME is the altered composition and architecture of the tumor ECM. Elevated secretion of collagen types I, II, III, V, and IX, along with hyaluronic acid, increases ECM stiffness. Collagen fibers transition from a relaxed, parallel arrangement to a rigid, radially oriented configuration, forming tracks that facilitate tumor cell migration [16,17,18,19,20,21]. Cancer-associated fibroblasts (CAFs) drive this excessive collagen deposition, which is further reinforced by the stiffness of the matrix itself—a feedback loop enhanced by the cross-linking enzyme lysyl oxidase (LOX) [16,18].

This progressive stiffening induces additional pathological features. Hypoxic tumor cores arise, fueling angiogenesis with irregular and leaky vasculature [21,22], shifting metabolism toward lactate production [18], and enhancing metastatic potential. Concurrently, altered interstitial fluid flow and shear stresses further modulate cellular proliferation and intravasation, promoting dissemination [20,21,23].

**Figure 1:**
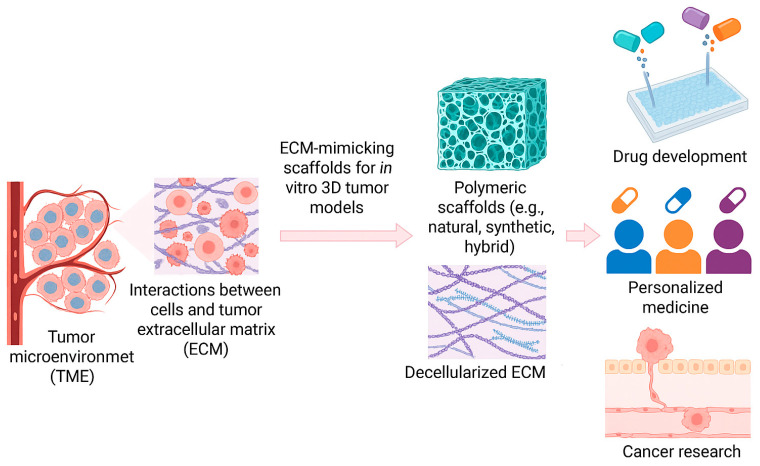
Schematic overview showing biomimetic scaffold strategies to simulate the complexity of the tumor microenvironment and their applications in basic cancer research, clinical use, and drug development.

Taken together, these observations illustrate that the TME is not merely a passive scaffold but a dynamic, self-reinforcing system that shapes tumor evolution and therapeutic resistance. However, despite extensive characterization, translating these insights into experimental models remains challenging. Accurately reproducing TME biochemical gradients, ECM stiffness, and fluid dynamics in vitro is critical for developing physiologically relevant tumor models capable of predicting clinical outcomes (Figure 1). In this context, the TME serves not only as a subject of description but also as a foundation for advancing next-generation culture systems.

## 3. The Importance of Tumor ECM

Although the TME consists of both cellular and acellular components that differentially drive tumor proliferation and differentiation, the acellular compartment, namely the tumor ECM, has emerged as a central determinant of cancer behavior. Far from being an inert scaffold, the ECM actively regulates cell migration, invasion, and therapeutic response, making it a critical element to replicate in vitro.

As early as 1980, Vlodavsky et al. [19] demonstrated the relevance of the ECM to tumor behavior by culturing human hepatocellular carcinoma and Ewing’s sarcoma cells on a healthy ECM produced by bovine corneal endothelial cells. Cells grown on this ECM formed clusters, exhibited metastatic-like migration and displayed markedly faster growth than cells cultured on plastic. Although still 2D, this model underscored the necessity of accurately reproducing the tumor’s native ECM environment to achieve physiologically relevant outcomes.

Before addressing the engineering of ECM surrogates, it is important to clarify how biomimetic substrates underpin different types of in vitro tumor models. The need for systems that capture in vivo tumor dynamics has led to a diverse set of model platforms. DePalma et al. [24] proposed a workflow for constructing 3D in vitro models, beginning with the identification of key ECM components and cell types within the TME. This step allows investigators to simplify the model while preserving essential features, such as omitting type I collagen for brain tumor models, where it is minimally present, and to select an appropriate modeling approach tailored to the research question.

Based on Karami et al. [25], Jouybar et al. [26], and Ravi et al. [27], the major model types can be summarized as follows, each highlighting distinct ways in which the tumor ECM can be mimicked or investigated:

- Spheroids: self-assembled cell aggregates (often via the hanging-drop technique) that recapitulate aspects of cell−ECM interaction. They can be scaffold-free (without biomimetic support structures) or scaffold-based. Their hypoxic cores and cell-derived ECM provide insights into tumor-like gradients and matrix remodeling, though their size and compositional control remain limited;
- Organoids: self-organized, 3D cell aggregates, derived from primary tumor tissue samples, stem cells, or induced pluripotent stem cells. The use of hydrogels as ECM substitutes allows for the study of diffusion, migration, and drug response in a 3D context. Despite their advantages over 2D cultures, their lack of intrinsic vasculature and missing TME elements still limit longevity and physiological fidelity;
- Transwell models: multi-compartment systems separated by semi-permeable porous membranes, enabling the study of gradients, migration, and tumor–endothelial interactions under controlled conditions;
- Microfluidic/organ-on-a-chip models: miniaturized systems with micrometer-scale channels simulating fluid flow and shear stress. These features, absent in static spheroid or organoid models, make them powerful tools for studying tumor proliferation, invasion, and drug response under physiologically relevant flow conditions. Integrated sensors and transparency facilitate real-time monitoring and multiplexed analysis. Specifically, in the context of cancer research, they are referred to as tumor-on-a-chip or cancer-on-a-chip models. These highly versatile models are generally based on a hydrogel scaffold, confined within the chip and connected to microchannels. Endothelial cells can be added to form monolayers lining the membranes that separate the different chip chambers. Additionally, the hydrogel scaffolds can be molded against temporary templates to create indentations that replicate endothelial lumens, aiding in the study of tumor invasiveness. The primary limitation of this model is the complexity of miniaturization;
- Bioprinted models: additive manufacturing of cell–ECM mixtures (“bioinks”) to create complex, crosslinked 3D architectures with tunable stiffness and composition. This approach promises unprecedented control over geometry and matrix properties but remains constrained by bioink availability, which may not be compatible with all types of 3D printers.

All these platforms underscore a central theme: without an ECM-mimicking scaffold that replicates the biochemical, mechanical, topographical, and geometric cues of the tumor ECM, it is impossible to achieve physiologically relevant models. Beyond biocompatibility, these scaffolds must actively support tumor cell proliferation and respond to specific chemical and mechanical stimuli associated with the tumor type under investigation.

By framing the tumor ECM as an active player rather than a passive background, current research is shifting toward models that integrate tunable stiffness, cell-matrix signaling, and fluid dynamics. These efforts provide the foundation for next-generation platforms capable of recapitulating not only tumor architecture but also the evolving microenvironment that drives malignancy.

## 4. Tumor ECM Engineering

In the last decades, the advent of tissue engineering paved the way for true ECM engineering: by precisely controlling chemical, mechanical, and topographical properties of biomaterials-based scaffolds, it is possible to create biomimetic substrates that support and promote cell proliferation and differentiation [28,29].

This section will discuss how tissue engineering principles can be applied to in vitro tumor modeling. In particular, the focus will be on tumor modeling through biomimetic scaffolds. Following a brief introduction to the concept of biomimetic scaffolds, the various currently available types and relevant examples will be presented.

### 4.1. Biomimetic Scaffolds

A scaffold is a support structure for cell adhesion and proliferation, leading to tissue growth [30]. It is a key element in tissue engineering, designed to develop fully differentiated tissues and organs. The scaffold acts as an artificial replacement of the ECM of native tissues. Therefore, the goal of a biomimetic scaffold is to replicate, as close as possible, the ECM characteristics of the target tissue and, in the present context, to model the TME [31].

Although each tumor has its own specific TME, the general characteristics of a scaffold for tumor modeling should include the following:

- Biocompatibility of chosen materials;
- Stiffness: The tumor ECM is stiffer than healthy ECM [32,33,34]. Scaffolds should have an elastic modulus similar to that of the tumor ECM [17]. Thus, the selected biomaterials should either be intrinsically stiffer than healthy tissue or be modifiable through crosslinking techniques to achieve the desired stiffness;
- Porosity and microarchitecture: The scaffold should present an interconnected pore network that mimics the natural ECM;
- Composition: It should replicate the chemical composition of the tumor ECM. At a minimum, it must include fundamental components for matrix-tissue integration, such as collagen, as well as other elements like hyaluronic acid or hydroxyapatite, for example, in an osteosarcoma model [35].

Additionally, another essential requirement is the ability to incorporate biochemical signals, such as recognition peptide sequences or growth factors like VEGF, to replicate, as closely as possible, the ideal tumor proliferation environment [17].

This set of desired properties can be achieved through the appropriate selection of biomaterials used in scaffold fabrication, considering the manufacturing, crosslinking, and functionalization techniques [17,36].

Over the last years, several scaffold-based in vitro tumor models have been developed. In the Section 4.2, we will discuss various examples of scaffolds used in tumor modeling, categorized according to the type of biomaterial selected.

### 4.2. Scaffold Typologies

#### 4.2.1. Natural Scaffolds

Natural biomaterials, once primarily used in tissue engineering, are increasingly exploited as functional platforms for predictive oncology models and drug discovery [35]. Their intrinsic bioactivity and resemblance to the ECM make them attractive for developing 3D tumor models that better replicate the TME than conventional 2D cultures. In this section, we highlight how they are engineered into 3D scaffolds for anticancer drug testing, radiotherapy evaluation, and phenotypic screening, and how their design can be further advanced to support high-throughput and personalized medicine.

Collagen, particularly type I, is one of the most widely used biomaterials for the fabrication of biomimetic hydrogels in the context of in vitro tumor models, to examine cancer cell invasion but also to predict radiotherapy and chemotherapy response. As a fundamental structural protein of the ECM, it promotes cell proliferation and tissue growth. Through chemical, thermal, or enzymatic crosslinking procedures, its stiffness can be regulated, yielding substrates reproducing a range of tumor tissue mechanics [20]. It naturally undergoes proteolytic degradation by collagenases, which allows for the in vitro study of substrate remodeling by tumor mass. However, this enzymatic degradation makes it unsuitable for long-term studies. Other limitations of this biomaterial are batch variability; a relatively low Young’s modulus that can be improved through crosslinking; processing difficulties; intense bioactivity, which inevitably alters cell evolution; and the degradation of adhesion sequences following processing treatments. Therefore, collagen is often used in combination with other natural biomaterials, such as alginate or chitosan.

Jia et al. [37] demonstrated that 3D collagen-based scaffolds markedly enhanced the clonogenicity, spheroid formation, and chemoresistance of glioma cells compared with conventional 2D cultures. Using bioinformatic analyses, the authors identified 77 genes commonly associated with both 3D culture and drug response, enriched in pathways related to stress responses, DNA damage and repair, and drug metabolism. Hub genes included AKT1, ATM, CASP3, CCND1, EGFR, PARP1, and TP53, which were predicted to be regulated by specific microRNAs. Collectively, their findings highlight 3D collagen scaffolds as physiologically relevant platforms for investigating glioma stemness and chemoresistance and for identifying potential diagnostic and therapeutic targets.

**Figure 2:**
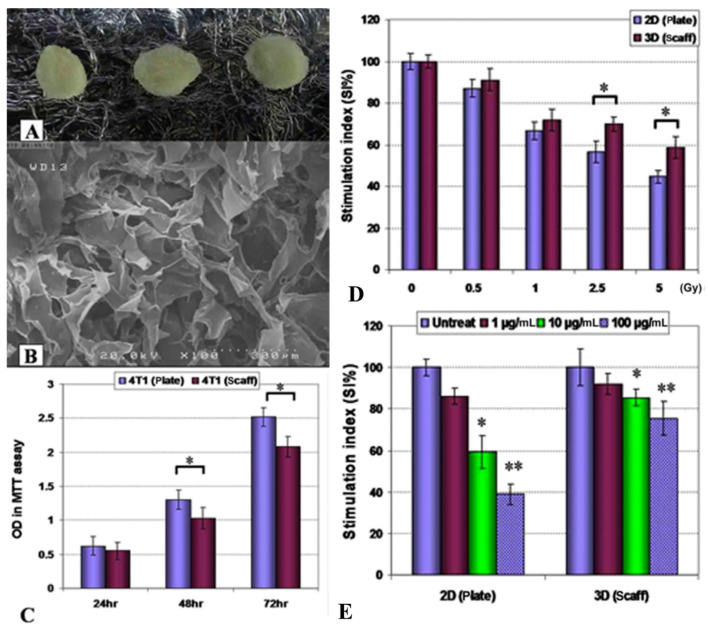
Macroscopic (**A**) and scanning electron microscopy (**B**) images of collagen/chitosan scaffolds. (**C**) Proliferation of 4T1 tumor cells on 3D scaffolds and under 2D culture conditions. After 48 and 72 h, cells exhibited significantly reduced growth on 3D scaffolds (*p* < 0.05). 4T1 cell viability following X-ray irradiation (0.5–5 Gy) (**D**) and exposure to 4-hydroxycyclophosphamide (**E**) (1–100 µg/mL) was assessed in 2D and 3D cultures. Cells grown on 3D scaffolds showed increased resistance to both high-dose X-ray irradiation (2.5–5 Gy) and 4-hydroxycyclophosphamide treatment compared to 2D cultures (*p* < 0.05). OD—optical density; MTT—3-(4,5-dimethylthiazol-2-yl)-2,5-diphenyltetrazolium bromide; scaff—scaffold; *, *p* < 0.05; **, *p* < 0.01. Reproduced from [38], an open-access article distributed under the terms of the Creative Commons Attribution License (CC BY).

In their study, Mahmoudzadeh and Mohammadpour [38] combined type I collagen with chitosan, a polysaccharide obtained by the deacetylation of chitin (found naturally in the shells of crustaceans), to create scaffolds for 3D in vitro tumor models. The produced porous scaffold was used to culture 4T1 breast cancer cells. These cells, grown in a 3D setting, were then subjected to X-ray radiation therapy and various anticancer drugs to determine their response (Figure 2). Compared to 2D cultures, the proliferation rate was slower and more consistent with the dynamics observed in patients. There was also greater resistance to both radiation therapy and chemotherapy as a consequence, respectively, of (i) the formation of tumor cell clusters that reduces the exposure of tumor cell to irradiation and (ii) the slower diffusion of drugs through the tumor mass. When the tumor cells, cultured on the 3D scaffold and subjected to radiation, were injected into rat models, a reduced immune response was observed, unlike in cells cultured on 2D plates and irradiated. This demonstrated that the results obtained from 3D cultures are more reliable in predicting in vivo behavior. Such models illustrate how natural polymers can support functional anticancer screening, enabling evaluation of treatment efficacy and tumor-stroma interactions under physiologically relevant conditions.

Horst et al. [39] developed interpenetrating polymer networks (IPNs) based on collagen−alginate and collagen−agarose to study epithelial ovarian cancer. The presence of two plant-derived sugars (one from brown algae and the other from *Rhodophyta* algae) provides structural rigidity to the scaffold, as they are not enzymatically degraded in the body. Additionally, the microarchitecture, consisting of an interconnected network with an adjustable elastic modulus via crosslinking, allows for the replication of the structural organization of the tumor ECM, providing both mechanical and biochemical stimuli to the cells being studied. In cell viability tests with OVCAR3 cells, Horst et al. observed over 80% viability in the first 48 h of culture. When testing the migration tendency of cells encapsulated in the IPNs hydrogel, a slight increase in migration was observed. Furthermore, cells cultured on stiffer scaffolds exhibited a greater migration tendency, similar to cells grown on culture plates (E = 10,000 kPa). Notably, these cells maintained their migration differences even after being removed from their IPNs, suggesting a form of “memory” regarding the substrate they were cultured on. This highlights the critical role of the ECM in epithelial−mesenchymal transition (EMT), a key process in tumor formation, where epithelial cells acquire embryonic/stem-like properties.

Another example of a collagen−alginate combination is the oxidized alginate and porcine liver collagen hydrogel proposed by Li Y. et al. [40]. The oxidation of alginate aldehydes allows for the formation of binding sites that are otherwise absent, imparting some bioactivity to the alginate component. The resulting hydrogel substrate was sufficiently rigid and non-immunogenic thanks to the presence of alginate, while still bioactive thanks to collagen. Encapsulating MGC-803 human gastric carcinoma cells in these ECM droplets led to increased proliferation, enhanced migration tendency, and greater expression of EMT-associated biomarkers, closely replicating in vivo observations. Additionally, cell morphology changed post-encapsulation, with increased polarization along the axis and the presence of pili at the extremities of this elongated axis—contrary to what is observed in 2D cultures.

Beyond collagen, another widely investigated natural polymer is gelatin, which is derived from collagen denaturation. As a degradation product, gelatin retains the same bioactive properties of collagen, though it consists of shorter and more heterogeneous single chains. The advantages of using gelatin over collagen in tissue engineering include lower costs, ease of processing, and various crosslinking methods to enhance its elastic modulus and viscosity. In their study, Wu et al. [41] investigated gelatin-based scaffolds to replicate the TME of esophageal squamous carcinoma stem cells. Their primary focus was substrate viscosity, which plays a crucial role in tumor tissue proliferation. More viscous scaffolds exhibited higher cell viability, larger colonies, and an increased survival rate in vitro, along with greater expression of pluripotency-related genes. When tumor cells from different scaffolds, 2D culture, and Matrigel™ were injected into mice, those from viscous scaffolds showed the highest tumor growth rate. Overall, developed scaffolds increased survival and stemness marker expression that are relevant readouts for testing agents targeting cancer stem cell populations.

Pele et al. [42] developed an innovative cancer-on-a-chip model using hybrid egg white/gelatin hydrogels to study pancreatic ductal adenocarcinoma. Compared with conventional collagen I scaffolds, these hydrogels provided greater mechanical stability, tunable stiffness, and a distinct globular nano-topography that supported the growth of large multicellular tumor structures over prolonged culture. Within microfluidic devices, PANC-1 cells formed both spheroids and grape-like aggregates that actively secreted ECM components and established strong cell–cell and cell–matrix adhesions, indicating enhanced mechanotransduction and mimicking in vivo tissue organization. By integrating a dual-chamber system for co-culture of PANC-1 cells with fibroblasts, the model enabled simultaneous analysis of angiogenic factor secretion, revealing complex cross-talk between tumor and stromal compartments during early PDAC development. This work highlights how the integration of biomimetic scaffolds in microfluidic platforms can address key limitations of traditional 3D cultures by combining defined mechanical cues with physiologically relevant biochemical environments, thereby offering powerful tools to investigate tumor progression, angiogenesis, and mechanobiology.

Gelatin can be further modified to obtain gelatin methacrylate (GelMA), which is synthesized through methacrylic anhydride modification [43]. Being derived from gelatin, which itself is a collagen hydrolysis product, GelMA contains arginine-glycine-aspartic acid (RGD) sequences, essential for cell adhesion, as well as recognition sites for metalloproteinases involved in substrate remodeling. The introduction of methacrylic groups enables photo-crosslinking, allowing the control of hydrogel topology. In their study, Kaemmerer et al. [44] developed a GelMA-based hydrogel for encapsulating ovarian cancer cells (OV-MZ-6). Analyzing various hydrogels with different polymer concentrations, they found that a 5% *w/v* polymer concentration was optimal, promoting cell proliferation and viability up to 21 days, highlighting their potential for medium-term drug testing and screening of microenvironment-modifying agents. Additionally, the incorporation of ECM components like hyaluronic acid and laminin-411 into GelMA hydrogels increased stiffness and pore size, enhancing cell proliferation and gene expression. This hybrid approach allows for highly customizable biomaterials that can replicate a variety of tumor ECM substrates. Future developments may integrate GelMA scaffolds with microfluidic systems and high-content imaging to enable high-throughput evaluation of therapeutic combinations.

Pamplona et al. [45] presented a versatile platform of photocrosslinked GelMA hydrogels designed to encapsulate colorectal (HCT-116) and pancreatic (MIA PaCa-2) cancer cells. By modulating UV curing times, the authors achieved a broad and reproducible range of stiffness values (0.16–4.8 kPa) that closely mimicked the mechanics of both healthy and tumor colon and pancreatic tissues. Atomic force spectroscopy confirmed fine-scale tunability at the microscale, and real-time live-cell imaging revealed distinct behaviors: HCT-116 cells formed size-dependent clusters influenced by matrix stiffness, whereas MIA PaCa-2 cells proliferated without significant aggregation. Notably, softer hydrogels promoted larger cell aggregates and greater migration permissiveness, while stiffer matrices constrained cluster size, providing an in vitro model of tumor progression under variable mechanical conditions. This work underscores the potential of GelMA-based hydrogels to systematically investigate how matrix stiffness and crosslinking chemistry regulate tumor cell growth, migration, and mechanotransduction, thereby offering an adaptable 3D scaffold system for colon and pancreatic cancer research.

Monette et al. [46] systematically evaluated how the intrinsic properties of scaffold materials modulate osteosarcoma cell behavior within 3D culture systems. Using three different natural matrices (collagen I hydrogel, gelatin microribbons, and GelMA), they demonstrated that scaffold composition strongly influences cell morphology, proliferation, and gene expression profiles, ultimately shaping the tumor-like phenotype of osteosarcoma cells. In particular, natural scaffolds such as collagen- and gelatin-based matrices supported enhanced cell–matrix interactions and more physiologically relevant ECM remodeling compared with their synthetic counterparts. This biomimetic environment also altered drug sensitivity, with osteosarcoma cells cultured on natural scaffolds displaying differential responses to standard chemotherapeutics relative to 2D or synthetic 3D conditions. These findings underscore the critical role of scaffold material selection in recapitulating key aspects of the TME, suggesting that natural biomaterials may offer a more predictive platform for studying malignancy progression and therapeutic efficacy in vitro.

Other notable materials used in the fabrication of biomimetic scaffolds include silk fibroin and amyloid fibrils. Silk fibroin is a protein produced by certain spiders or obtained from the cocoons of Bombyx mori silkworms. This biopolymer exhibits excellent mechanical properties, good permeability to water and oxygen, high biocompatibility, and anti-inflammatory properties that promote cell adhesion. In the context of tissue engineering, it has been primarily utilized for applications related to skin, bone, cartilage, and neural tissue. In their study, Kumar and Packirisamy [47] used fibroin combined with alginate to fabricate porous scaffolds through lyophilization for in vitro cancer models. Beyond providing mechanical support and a porous morphology, these scaffolds can be functionalized to deliver biochemical stimuli to cells both during culture and during the scaffold fabrication process itself. The use of these alginate−fibroin beads for culturing A549 lung cancer cells revealed increased resistance to anticancer agents compared to 2D monolayer cultures. The presence of fibroin enhanced cell adhesion, survival, and viability compared to cells cultured on pure alginate-based particles.

The study by Singh et al. [48] reported the applications of amyloid fibrils. Amyloid refers to fibrous protein deposits found in organs and tissues, which are insoluble and organized into a β-sheet structure. Amyloid is often associated with various pathologies, such as amyloidosis, characterized by the accumulation of misfolded protein fibrils. In this study, amyloid fibrils exhibited cytocompatibility and bioactive properties, which were leveraged to develop thixotropic hydrogels—materials that become less viscous when subjected to shear stress—capable of promoting cancer cell proliferation. This particular viscoelastic property facilitates cell encapsulation. Specific sequences within the fibrils stimulate integrin receptors, enhancing cell adhesion and the formation of focal adhesions within the cytoskeleton. Singh et al. employed these scaffolds as substrates for various tumor cell lines, including breast cancer (MCF7, MDA MB 231), hepatocellular carcinoma (HepG2), cervical cancer (HeLa), and lung cancer (A549). All cell lines exhibited the formation of cell aggregates on the scaffold. Notably, the cells were able to migrate and fuse on the substrate, leading to the formation of aggregates that progressively grew into more compact and spherical structures. Cell viability assessments indicated the development of a hypoxic core within larger spheroids, consistent with in vivo observations. Biomarker analysis revealed an upregulation of EMT markers, while tumor suppressor genes such as *CCND2* and *CD24* were downregulated. This biomarker overexpression persisted over prolonged culture periods, with increased expression of markers like VEGF observed up to the seventh day of incubation. Consequently, amyloid fibrils represent a promising approach for the development of bioactive scaffolds for various tumor cell lines. Additionally, their thixotropic nature makes them particularly useful for replicating dynamic cellular environments, with specific biomechanical characteristics. The reported persistent upregulation of EMT markers and VEGF in multiple tumor cell lines grown on amyloid scaffolds suggests their use as predictive models of angiogenesis and metastasis during therapy screening.

Another widely used material in tissue engineering and in vitro tumor modeling is Matrigel^TM^, derived from the ECM of Engelbreth-Holm-Swarm sarcoma cells in mice. As a decellularized tumor-derived matrix, Matrigel^TM^ is rich in collagen IV and laminins, making it suitable for replicating the chemical composition of the ECM. In their study, Li N.T. et al. [49] developed a tumor organoid culture platform based on cellulose scaffolds coated with a hydrogel composed of 75% collagen and 25% Matrigel^TM^. This specific ratio facilitated the formation of non-aggregated clusters and ensured a uniform cell distribution within the hydrogel. The presence of the cellulose “sheet” provided structural support to the hydrogel, protecting it from degradation. Furthermore, stabilizing the hydrogel on the cellulose scaffold allows for the development of thicker cultures, as a hydrogel composed solely of collagen could be deformed by tumor cell growth. This approach enables the replication of larger cultures that better mimic parenchymal tissue. Such hybrid scaffolds provide improved culture longevity and can be adapted to screen drugs over extended time frames, including evaluation of combination therapies and late-emerging resistance. One limitation of this model, despite its simplicity and efficiency for evaluating organoid growth and viability, is its inadequacy for mechanobiology studies, as its porosity may restrict cellular contraction.

In addition to proteins, polysaccharides of animal origin have been also used. Porous chitosan–hyaluronic acid scaffolds, as described by Florczyk et al. [50], offer a highly biomimetic platform to replicate the ECM of glioblastoma. These scaffolds are fabricated by forming a polyelectrolyte complex between chitosan and hyaluronic acid, followed by lyophilization, resulting in a 3D porous network with pore sizes ideal for tumor cell colonization. When U-118 MG glioblastoma cells were cultured within these scaffolds, they formed compact spheroids and exhibited enhanced stem-like characteristics, including elevated expression of CD44, Nestin, Musashi-1, GFAP, and HIF-1α, compared to 2D cultures. Importantly, the 3D microenvironment promoted increased invasiveness and chemoresistance, linked to the upregulation of the ABCG2 efflux transporter. These effects are attributable to the scaffold’s biochemical mimicry (hyaluronic acid recreating glycosaminoglycan-rich ECM) and mechanical cues, since scaffold stiffness and porosity modulate cellular behavior. Taken together, chitosan–hyaluronic acid scaffolds demonstrate how natural biomaterials, when engineered into 3D architectures, can effectively reproduce key elements of the tumor microenvironment—supporting malignant phenotypes and providing a more physiologically relevant in vitro platform suitable for mechanistic studies and drug screening in glioblastoma research. The demonstrated capability of these scaffolds to promote spheroid formation, stem-like phenotypes, and chemoresistance makes them valuable for testing efflux transporter inhibitors or radiosensitizers.

Wang et al. [51] have shown that 3D porous scaffolds composed of chitosan alone or chitosan–hyaluronic acid blend robustly enrich glioma stem cell-like populations compared to conventional 2D cultures. When U-87 or U-118 glioma cells were seeded on these scaffolds, they formed compact spheroids and exhibited significantly higher expression of glioma stem cells-related markers, including CD133 and SOX2, along with enhanced levels of EMT genes such as Snail and N-cadherin. Notably, the chitosan–hyaluronic acid scaffolds led to even greater enrichment than chitosan-only scaffolds, indicating that hyaluronic acid incorporation potentiates stemness, likely through its native interactions with CD44 and RHAMM receptors. Importantly, cells cultured on chitosan–hyaluronic acid scaffolds displayed higher in vivo tumorigenic potential in xenograft models, producing larger and faster-growing tumors than their 2D or chitosan-cultured counterparts. These findings emphasize that the chemical composition of natural scaffolds is a critical determinant of microenvironmental cues: the combined use of chitosan and hyaluronic acid not only enhances spheroid formation but also supports EMT processes and tumor initiation capacity. Consequently, chitosan–hyaluronic acid scaffolds represent a powerful tool for enriching glioma stem cells in vitro, offering a physiologically relevant platform for investigating cancer stem cells biology and preclinical screening of anti-glioma therapies.

Gebeyehu et al. [52] developed polysaccharide-based hydrogels as bioinks for extrusion bioprinting of tumor models, demonstrating how natural polymer systems can closely mimic the ECM in vitro. Using a xeno-free VitroGel platform modified with RGD motifs, they achieved high printability, structural fidelity, and >90% post-printing cell viability across multiple cancer cell lines. The resulting 3D scaffolds supported rapid spheroid formation of patient-derived non-small-cell lung cancer (NSCLC) cells within seven days and recapitulated key stromal features, including ECM stiffness and cell–cell adhesion markers (E-cadherin and vimentin). Compared with conventional 2D cultures, cells in the 3D hydrogel scaffolds exhibited markedly increased resistance to standard chemotherapeutics such as docetaxel, doxorubicin, and erlotinib, highlighting the impact of a natural ECM-like environment on drug responses. This study underscores the promise of bioprinted natural hydrogels as tunable and physiologically relevant scaffolds for building TME models suitable for high-throughput drug screening.

Moving to polysaccharides of plant origin, alginate is one of the most investigated in the fabrication of biomimetic scaffolds for tumor engineering. Estrada et al. [53] developed a 3D co-culture model based on long-term alginate microencapsulation to mimic key features of the breast tumor microenvironment during disease progression. In this system, MCF-7 breast cancer cells were co-cultured with human fibroblasts in spherical alginate microcapsules under dynamic suspension conditions, enabling prolonged culture and physiological cell–cell interactions over 15 days. The fibroblasts localized to the periphery of the tumor spheroids, forming a stromal-like layer that actively secreted collagen and pro-inflammatory cytokines, thus creating a compartmentalized tumor-stroma structure. Notably, the tumor cells underwent progressive phenotypic changes, including loss of estrogen receptor expression, downregulation of membrane E-cadherin, loss of polarity, and increased angiogenic potential—all consistent with tumor progression in vivo. These transitions were absent in monocultures, highlighting the essential role of stromal–epithelial interaction in driving malignancy. The model also demonstrated enhanced collective migration and features of drug resistance, making it suitable for long-term therapeutic testing. Overall, the study shows that natural hydrogels like alginate, when integrated into 3D co-culture systems, can effectively recreate dynamic aspects of tumor biology, providing a scalable, time-resolved platform for investigating tumor-stroma crosstalk and phenotypic plasticity under physiologically relevant conditions.

De et al. [54] designed a micron-scale alginate-based 3D scaffold to closely replicate tumor architecture and enable real-time monitoring of encapsulated cell behavior. These micro-scaffolds incorporated carbon-dot pH nanosensors, allowing continuous, non-invasive tracking of the tumor microenvironment pH without the need for endpoint assays. Structurally, the scaffolds exhibited a heterogeneous, core–shell architecture that leads to the formation of a hypoxic core within 96 h, replicating in vivo tumor oxygen gradients. Porosity and density were tunable, enabling adjustment of ECM mimicry to specific experimental needs. When loaded with hepatocellular carcinoma cells, the scaffolds supported robust growth, enhanced expression of liver-specific functional markers, and physiologically relevant non-uniform molecular diffusion, mimicking treatment penetration challenges in solid tumors. The developed platform demonstrated how natural polymers like alginate, enhanced with smart nanosensors, can effectively recapitulate key biophysical and biochemical features of solid tumors. The integration of sensing functionalities and adjustable architecture makes these alginate-based micro-scaffolds promising for dynamic 3D cancer modeling, real-time drug screening, and studies of tumor metabolism under physiologically relevant conditions.

Rizzo et al. [55] reported an alginate-based 3D scaffold system functionalized with silica microparticle ratiometric pH sensors to map extracellular acidity within tumor–stroma co-cultures. Exploiting the biocompatibility, transparency, and tunability of alginate microgels, the authors microencapsulated pancreatic adenocarcinoma cells and pancreatic stellate cells alongside fluorescent pH probes, achieving high viability and nutrient diffusion throughout the constructs. This platform enabled real-time confocal imaging of spatial and temporal pH gradients at single-cell resolution, revealing distinct acidification profiles between mono- and co-cultures that reflected tumor–stroma metabolic crosstalk. By providing a natural hydrogel environment combined with an integrated sensing system, the alginate microgels closely reproduced key microenvironmental features of pancreatic tumors, including desmoplastic stroma and acidic gradients, thereby offering a robust and non-invasive tool for investigating tumor metabolism and drug response in physiologically relevant 3D conditions.

In summary, natural biomaterials offer a valuable resource for developing biomimetic scaffolds for TME modeling, thanks to their high biocompatibility and intrinsic bioactivity. Their evolution from static matrices to dynamic, sensor-integrated 3D systems signals a shift toward drug discovery applications. Incorporating natural scaffolds into automated, high-throughput microfluidic systems could enable simultaneous testing of multiple drug candidates under physiologically relevant conditions. Combining these hydrogels with real-time biosensors (pH, oxygen, and metabolic markers) may facilitate dynamic monitoring of tumor responses to therapy, bridging the gap between preclinical testing and clinical outcomes. Natural scaffolds are no longer merely materials of biological origin but have become platforms for predictive oncology, supporting anticancer drug screening, toxicity testing, and mechanistic studies. By refining their mechanical properties, incorporating sensing capabilities, and integrating them into organ-on-chip and high-content imaging systems, these biomaterials can play a pivotal role in the next generation of in vitro malignancy models.

On the other hand, natural biomaterials also present several limitations. Since they are derived from natural sources, extraction methods do not always preserve the properties of the native component. A notable example is collagen, which loses its mechanical properties during extraction and requires cross-linking. Additionally, the heterogeneity of natural sources, along with the need for cross-linking processes, leads to variability between different batches of the same natural biomaterial, ultimately affecting the reproducibility of the scaffolds discussed in this section. Finally, although cross-linking techniques exist for nearly all types of natural biomaterials, their effects on cross-linking degree and mechanical properties are often unpredictable, making it challenging to achieve the desired mechanical characteristics for a given scaffold.

Consequently, there is a growing need to work with more customizable biomaterials, where density, porosity, topography, mechanical properties, and degradation kinetic can be precisely controlled. In this regard, synthetic biomaterials appear very interesting. In the Section 4.2.2, we will discuss scaffolds made from synthetic biomaterials, whose properties can be controlled and modulated and that can be extensively modified using various fabrication techniques.

#### 4.2.2. Synthetic Scaffolds

Synthetic biomaterials are laboratory-engineered polymers that allow precise control over their mechanical, geometric, and chemical properties. In the context of tumor modeling, their main advantage lies in their high reproducibility and tunability: stiffness, porosity, topography, and degradation kinetics can be adjusted to mimic the TME and to probe specific cellular pathways such as mechanotransduction. Commonly used polymers include polyglycolic acid (PGA), polylactic acid (PLA, with two variants: PDLA, obtained with both stereoisomers of lactic acid, and PLLA, containing only L-isomers), their copolymers (PLGA), polycaprolactone (PCL), polyvinyl alcohol (PVA), polyethylene glycol (PEG), polyethylene glycol diacrylate (PEGDA), and polyurethanes (PU) [56].

A pioneering example of synthetic biomaterials used for in vitro tumor models can be found in the study by Sahoo et al. [57]. This study proposed porous microparticles made of PLGA and PLA for studying breast cancer cells (MCF-7 cell line). The choice of biodegradable polyesters is functional in providing cellular support during tissue growth and ECM deposition, after which the biomaterial should ideally be resorbed at a degradation rate compatible with ECM deposition by the cells. To enhance biocompatibility, the surface hydrophilicity of the microparticles was modified during fabrication to promote cell adhesion by incorporating PVA into the internal matrix. PVA, being a highly hydrophilic synthetic polymer, enabled the formation of a hydrophilic core. Among the two proposed polyesters, PLA demonstrated superior interaction with PVA, forming an interconnected network with hydrophobic and hydrophilic regions, consequently enhancing cell proliferation and adhesion, while mimicking early ECM deposition during tumor formation. Cells cultured on this scaffold exhibited reduced apoptosis, increased receptor expression, and partial differentiation compared to those grown on 2D monolayers.

PLLA remains widely used for modeling stiff tissues. For instance, Lombardo et al. [58] used a porous PLLA scaffold fabricated through thermally induced phase separation (TIPS) to create an ideal support for culturing MDA MB 231 breast cancer cells. By optimizing the reaction time and temperature during the TIPS procedure, they identified an optimal fabrication protocol that produced an interconnected microporous network with the desired pore diameter. The introduction of an insulating polytetrafluoroethylene (PTFE) coating during the cooling phase resulted in PLLA scaffolds with a pore diameter of 40–50 μm, identified as ideal for promoting tumor proliferation and the formation of “stellate” cellular aggregates, a morphology characteristic of highly invasive cell lines. Additionally, pre-treating PLLA with type I collagen overcame the inherent lack of bioactivity in PLLA, providing a bioactive and biocompatible substrate for cell adhesion and growth.

**Figure 3:**
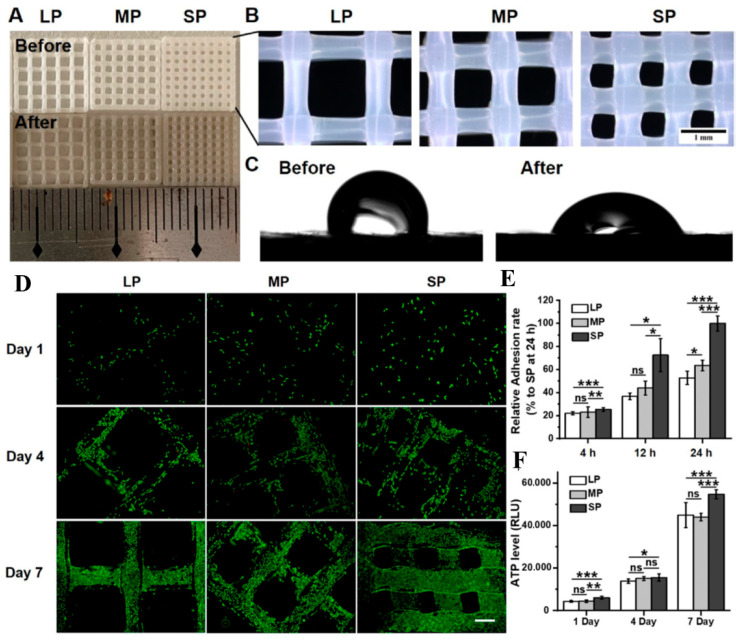
Photographs (**A**) and stereomicroscope images (**B**) of 3D-printed PLLA scaffolds with large (LP), medium (MP), and small (SP) pore sizes, illustrating morphological differences and surface architecture. (**C**) Water contact angle measurements before and after polydopamine (PDA) coating, indicating changes in surface hydrophilicity. (**D**) Live/dead fluorescent staining of MG-63 cells after 1, 4, and 7 days of incubation on the scaffolds (scale bar: 500 μm), demonstrating cell viability over time. (**E**) Quantitative analysis of cell adhesion at 24 h. (**F**) Cell proliferation assessed by ATP content, highlighting scaffold-dependent growth dynamics. *, *p* < 0.05; **, *p* < 0.01; ***, *p* < 0.001; ns, no significance. Reproduced from [59], an open-access article distributed under the terms of the Creative Commons Attribution License (CC BY).

Another application of PLLA in tumor modeling was reported in the study by Wang et al. [59], which aims to investigate the influence of cortical bone ECM properties on osteosarcoma progression and metastasis. This study sought to address the limitations of many natural hydrogel-based models in replicating the cortical bone matrix due to their low elastic modulus. PLLA was extruded, by 3D printing, into filaments to create a network with controlled porosity and interconnected pores, that better resembled the fibrous microarchitecture of the ECM, as well as similar stiffness as cortical bone. The effect of different pore sizes was investigated. To enhance bioactivity, the scaffolds were functionalized with polydopamine, increasing their overall hydrophilicity. Among the various fabricated samples, those with smaller pore sizes (d = 0.518 ± 0.015 mm) showed the most promising results in terms of osteosarcoma cell adhesion and proliferation (Figure 3). Moreover, PLLA scaffolds resulted able to more accurately replicate the in vivo bone TME in terms of cytoskeletal organization, energy metabolism, expression of ECM components, surface receptors, and growth factors. Furthermore, transcriptomic analysis reveals a high level of agreement in the KEGG pathway activation and signaling profiles, along with the identification of predictive biomarkers consistent with clinical data.

Polyurethane scaffolds have also been used for complex co-culture systems. Angeloni et al. [60] explored the use of polyurethane for scaffold fabrication to model bone metastases in breast cancer. After verifying the high porosity and interconnectivity of the structure, as well as its cytocompatibility, the scaffolds were seeded with adipose-derived stem cells (ADSCs), to generate a bone-like matrix before introducing breast cancer cells. These cells not only proliferated on the supporting structure but also differentiated into osteoblasts, producing their own bone matrix containing traces of calcium phosphate, osteopontin, and osteonectin. Following ADSC culture and differentiation, breast cancer cells of the MCF-7 cell line were seeded to establish a co-culture. Tumor cells exhibited a spherical morphology, distinct from the flattened, elongated shape of differentiated ADSCs, mimicking the spatial arrangement typical of metastatic tumor sites. The polyurethane used resulted sufficiently biostable to provide a long-lasting substrate, capable of maintaining its mechanical properties even after repeated compression cycles. This characteristic mimicked the cortical bone, which retains its porosity after mechanical loading. Moreover, produced scaffolds showed high water absorption and porosity, making them an ideal support for cell proliferation, migration, and nutrient diffusion. Additionally, the used polyurethane is an economical and easily processable material, making it an effective and accessible scaffold for replicating the bone microenvironment in 3D models. Furthermore, ADSCs can be readily harvested from patients, allowing for personalized studies. Overall, this approach replicated spatial arrangements of bone metastases and allowed analysis of cell–matrix interactions under mechanical loading, demonstrating the substrate’s long-term stability and capacity for high-throughput screening.

**Figure 4:**
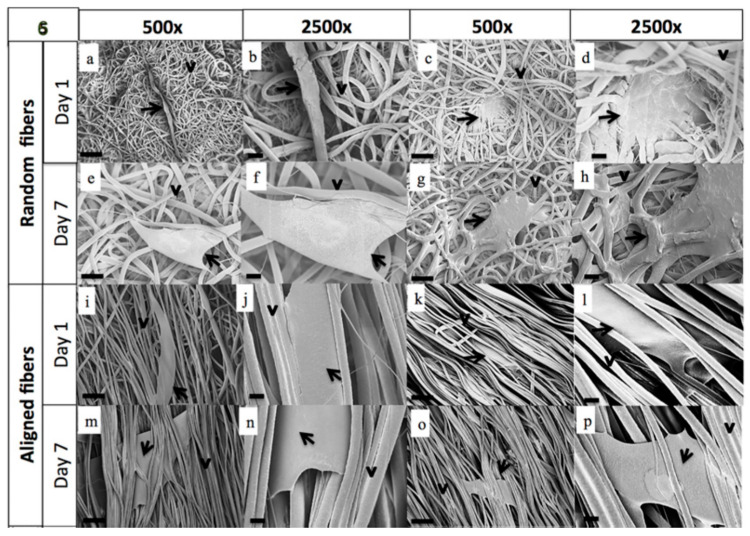
Scanning electron microscopy (SEM) images showing MDA-MB-231 cells cultured on fibrous scaffolds at day 1 and day 7. Arrows indicate the cell bodies, while arrowheads highlight the underlying scaffold fibers. Reproduced from [61], an open-access article distributed under the terms of the Creative Commons Attribution License (CC BY).

PCL was also used as scaffold material in tumor modeling. Guiro et al. [61] explored how electrospun PCL fibrous scaffolds with either random or aligned fiber orientations can influence breast cancer cell behavior and dormancy (Figure 4). These PCL scaffolds mimic the structural organization of collagen in the tumor ECM, offering a 3D environment superior to traditional 2D cultures. The authors induced chemoresistance in MDA-MB-231 and T47D cells using carboplatin, finding that resistant cells exhibited elevated expression of anti-apoptotic and stem-cell markers (Bcl-2, Oct-4, and Sox-2). When seeded onto fibrous scaffolds, MDA-MB-231 cells maintained stable cell numbers over seven days, unlike their rapid proliferation on tissue culture plastic. Notably, non-treated cells on scaffolds showed sustained cyclin D1 expression, indicating a shift away from proliferation typical of 2D culture. Chemoresistant cells retained dormancy-like behavior in both scaffold and plastic environments. Overall, their findings suggest that scaffold architecture, and not just composition, induced dormancy-like behavior and altered the expression of focal adhesion proteins (integrins, FAK), thus linking scaffold mechanics to treatment response. By recapitulating key physical and biochemical cues of the in vivo tumor niche, such PCL fibrous scaffolds provide a robust platform for studying drug resistance, tumor dormancy, and cancer stem cell dynamics in vitro.

Despite their highly tunable mechanical and degradation properties, low cost, and ease of fabrication into various forms, synthetic biomaterials have a major limitation: their inability to guide cellular responses. Since they do not contain naturally occurring ECM components, they lack specific signals that promote cell adhesion and proliferation. A promising strategy to overcome the intrinsic limitations of both natural and synthetic materials is their integration, aiming to achieve controlled mechanical and morphological characteristics combined with a high degree of bioactivity. The Section 4.2.3 discusses “hybrid” biomaterials, which aim to synergically integrate the properties of synthetic and natural biomaterials to create scaffolds that faithfully replicate the TME in terms of biomechanical, morphological, and biochemical characteristics. These next-generation hybrid scaffolds thus serve not only as 3D supports but also as precision tools for dissecting cell–matrix interactions, mechanotransduction pathways, and drug responses, directly aligning with the needs of anticancer drug discovery.

#### 4.2.3. Hybrid Scaffolds

In this section, the term “hybrid scaffolds” refers to scaffolds containing both synthetic and natural biomaterials, to effectively integrate the properties of each category. In the fabrication of hybrid scaffolds, a common approach involves combining a natural biomaterial with desirable bioactive properties but poor mechanical performance, with a synthetic biomaterial that enhances mechanical stability without compromising the bioactivity of the resulting material.

One of the most challenging natural biomaterials to process is type I collagen, a key ECM component widely used in tumor modeling. To control scaffold porosity, De Jaeghere et al. [62] developed hybrid hydrogels based on PLA modified with type I collagen. These scaffolds were seeded with cancer-associated fibroblasts (CAFs) to better replicate the peritoneal TME for metastasis studies. The presence of CAFs, which contribute to ECM remodeling and TME formation, promoted spheroid and cellular aggregate formation, while increasing the scaffold’s structural stiffness.

Inspired by the spongy, microporous structure of loofahs, combined with their excellent mechanical compression properties, Liu X. et al. [63] developed a sponge-like scaffold using polyethylene terephthalate (PET)-based spacer fabric for prostate cancer cell culture. A spacer fabric consists of two fabric layers separated by a layer of spacer yarn, which allows for a controlled interlayer distance. PET, a non-biodegradable polymer widely used in implantable prostheses such as hernia meshes and vascular grafts, was used to form a sandwich-like structure that provided structural support to collagen fibers, which were uniformly distributed within the scaffold. Swelling, sodium chloride diffusion, and cyclic compression tests demonstrated that this composite scaffold exhibited superior mechanical properties compared to both pure collagen hydrogels and PET sponges, maintaining a high elastic modulus and preserving porosity even in the presence of liquid absorption and under cyclic compression. The tunable pore size achieved during fabrication resulted optimal for both cellular adhesion and proliferation, as well as for substance transport and waste removal. The culture of DU 145 prostate cancer cells, known for their tendency to form spheroids, demonstrated increased proliferation and infiltration in hybrid scaffolds compared to pure control materials (PET sponge, collagen scaffold), along with a uniform distribution of cell aggregates within the scaffold, making it an excellent in vitro tumor model.

Similarly, with the aim of developing a microfibrillar scaffold that better replicates the TME architecture, Khatami et al. [64] fabricated a tubular scaffold composed of electrospun nanofibers with a gelatin core and a PLA coating. Electrospinning is a technique in which a biomaterial solution is held in a syringe with a metallic needle. The application of a high voltage between the syringe needle and a collector plate causes the biomaterial solution to be extruded in form of nanofibers, due to the applied electric field. These fibers were used to construct a tubular scaffold that better mimicked the shape of the mammary duct, where ductal carcinoma in situ typically proliferates. The presence of gelatin and PLA enabled the formation of hydrophilic and hydrophobic regions, effectively replicating the composition of the TME: gelatin provided a hydrophilic core, while PLA formed a hydrophobic outer shell. Scanning electron microscopy (SEM) images revealed MCF-7 cell proliferation both inside and outside the nanofibers, as well as ECM deposition by tumor cells, both indicative of successful cellular adhesion and proliferation.

Another example of hybrid scaffold, fabricated through 3D bioprinting, was reported by Cheng et al. [65]. This study aimed to provide an in vitro model for investigating breast cancer bone metastases, with a focus on the role of the vascular component in tumor cell migration. To accurately reproduce the conditions under investigation, a 3D bioprinted scaffold was designed. The scaffold was composed of three parallel-printed components: a porous section made of photocrosslinkable polyethylene glycol diacrylate (PEGDA) and GelMA hydrogel, to represent the TME of breast cancer; a tubular component, also made of photocrosslinkable (PEGDA) and GelMA, to simulate the vascular conduit; and a porous component composed of PEGDA and GelMA integrated with hydroxyapatite, to mimic the bone microenvironment. The selection of photocrosslinkable PEGDA and GelMA as the base for the bioink allowed for the creation of a biocompatible and bioactive scaffold, with highly controllable mechanical properties. The vascular portion of the scaffold was populated with human umbilical vein endothelial cells (HUVECs) to engineer a functional vascular conduit. This setup enabled not only the evaluation of angiogenesis stimulated by the tumor tissue, as the HUVECs successfully proliferated on the scaffold, but also the study of tumor cell migration driven by the presence of vascular tissue. This innovative model represents the first attempt in which a fundamental component of the TME—the vascular network—has been faithfully replicated, a critical factor in metastasis formation. The presence of a vascular component makes this model suitable for dynamic studies, allowing for further investigation into the relationship between tumor evolution and mechanical stimuli.

Rao et al. [66] developed a core–shell electrospun nanofiber platform to mimic the topographical cues of brain white matter tracts and dissect their influence on glioblastoma multiforme (GBM) cell migration. By using PCL shell to maintain consistent surface chemistry and independently varying the fiber core material—gelatin, poly(dimethylsiloxane) (PDMS), or poly(ethersulfone) (PES)—they generated aligned fibers with identical morphology but tunable stiffness. GBM cells displayed stiffness-sensitive behavior: single-cell shape (Feret diameter), migration speed, FAK activation, and MLC2 expression were all modulated by the core modulus, with intermediate stiffness (gelatin-PCL) yielding slower migration and reduced focal adhesion signaling. The platform was also employed to study chemical cues by incorporating hyaluronic acid, collagen, or Matrigel in the shell while preserving mechanical properties, revealing that hyaluronic acid significantly reduced migration rates. This hybrid scaffold strategy clearly distinguishes the contributions of mechanical stiffness and surface chemistry in GBM migration, replicating both the anisotropic topography of neural tracts and the complexity of the tumor microenvironment. The findings underscore the importance of mechanotransduction and biochemical signaling in driving invasion and suggest that such hybrid electrospun platforms are powerful tools to study malignancy behavior and potentially screen anti-migratory therapeutics in vitro.

Another example of hybrid scaffolds is represented by natural polymers combined with ceramic materials, such as hydroxyapatite and calcium phosphates, resembling the inorganic component of bone ECM. Zhu et al. [67] integrated hydroxyapatite into a chitosan-based hydrogel to model the bone ECM for studying breast cancer bone metastases. Using 10% hydroxyapatite nanoparticles resulted in the best balance of bioactivity and topographical properties. The incorporation of mesenchymal stem cells (MSCs) further modified the substrate to better mimic bone microenvironments, facilitating in vivo relevant observations.

Bassi et al. [68] engineered two complementary bone-mimicking hybrid scaffolds—a low-stiffness Mg-doped hydroxyapatite nucleated on self-assembling collagen fibers (MgHA/Coll, ≈31 kPa) and a high-stiffness sintered porous hydroxyapatite ceramic (≈1.8 GPa)—to recreate the diverse biophysical cues of the osteosarcoma stem-cell niche. Using sarcosphere culture to enrich cancer-stem-like cells from MG-63 and SAOS-2 lines, the authors compared cancer stem cells behavior on the two 3D scaffolds versus conventional 2D plastic. Both scaffolds preserved spheroidal morphology and markedly up-regulated stemness genes OCT-4, NANOG and SOX-2, with hydroxyapatite inducing up to ~40-fold increases in NANOG and OCT-4, and MgHA/Coll eliciting >4-fold rises in NANOG (*p* < 0.01). Niche-interaction transcripts (NOTCH-1, HIF-1α, and IL-6) were also significantly elevated, indicating active cancer stem cells—matrix crosstalk. Importantly, scaffold stiffness modulated responses: the softer MgHA/Coll preferentially boosted NOTCH-1/HIF-1α in MG-63 spheroids, whereas the stiffer hydroxyapatite accentuated NANOG in both lines. These findings show that combinatorial hybrid scaffolds capturing both chemical composition (collagen + hydroxyapatite) and mechanical heterogeneity can more faithfully replicate the osteosarcoma cancer stem cell niche, offering a predictive 3D platform to study tumor heterogeneity and screen anti-osteosarcoma therapies, thereby bridging the translational gap between in vitro testing and clinical outcomes.

Recently, Weng et al. [69] engineered a bioinspired composite hydrogel scaffold (GHP4a) composed of GelMA, hyaluronic Acid methacryloyl, and 4-arm PEGDA to replicate the ECM for colorectal cancer models. The GHP4a hydrogel exhibited biomimetic mechanical properties (600–700 Pa modulus), high porosity, and excellent biocompatibility, enabling Caco-2 cell encapsulation, spheroid formation, and long-term survival. Notably, cells cultured within GHP4a displayed enhanced anoikis resistance relative to conventional 2D cultures, linked to activation of integrin-mediated FAK and PI3K/Akt signaling and reduced caspase-mediated apoptosis. Transcriptomic, single-cell, and radiomic analyses confirmed that the hydrogel induced distinct molecular and structural phenotypes, recapitulating key aspects of the TME such as metabolic reprogramming, immune cell heterogeneity, and tissue stiffness. By closely mimicking the natural ECM and sustaining cell–cell and cell–matrix interactions, the GHP4a scaffold provides a physiologically relevant in vitro platform for investigating mechanisms of anoikis resistance and evaluating therapeutic interventions targeting metastatic progression.

The integration of biomaterials of different origins represents a promising strategy for developing comprehensive artificial TMEs with controllable physicochemical properties that closely resemble those of the native TME. The introduction of innovative fabrication techniques, such as bioprinting, enables the creation of complex microarchitectures that mimic the geometric, morphological, and topographical characteristics of the TME.

Although these scaffolds represent perhaps the best compromise for TME modeling, they still lack a certain degree of personalization, which is crucial for the development of in vitro models for personalized therapy. Therefore, in the Section 4.2.4, we will discuss a range of innovative scaffolds that are paving the way for patient-specific modeling (and potentially, personalized therapy): decellularized ECM (dECM)-based scaffolds.

#### 4.2.4. Alternative Scaffolds: Decellularized ECM (dECM)

As the name suggests, dECM is obtained by removing the cellular component from a specific tissue through chemical, physical, and enzymatic treatments, in order to obtain a matrix free of cellular components that can be used as support for cell culture without eliciting an immune response. dECM represents a promising class of scaffolds for in vitro tumor models, as they preserve the biochemical complexity, intrinsic bioactivity, and microarchitecture of the native tissue. Compared to synthetic or natural materials, dECM provides a microenvironment that more closely reflects the composition of the tissue of origin, enabling more physiologically relevant cancer models [70].

**Figure 5:**
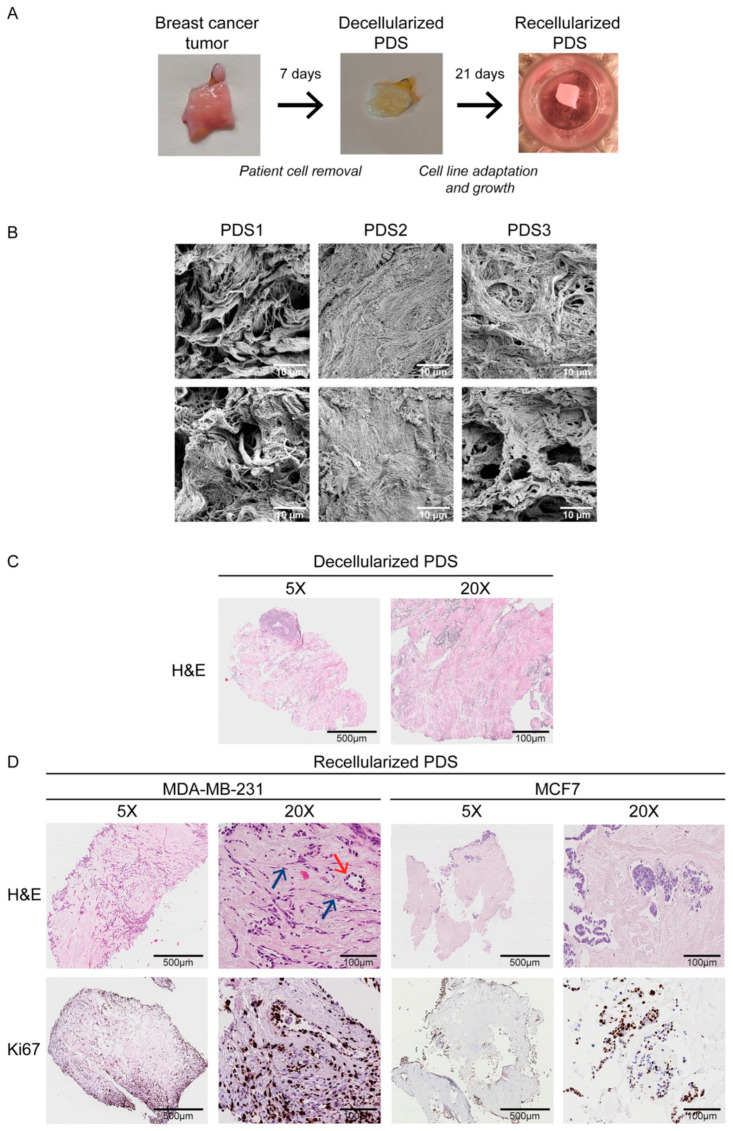
Decellularized, patient-derived breast cancer scaffolds (PDSs) provide a cell-free matrix that supports cellular infiltration and growth. (**A**) Schematic workflow of PDS production with representative images. (**B**) Scanning electron microscopy images of three decellularized PDSs at two locations highlights both intra- and inter-tumoral heterogeneity (scale bar: 10 μm). (**C**) H&E staining confirms the absence of cells while preserving the scaffold’s structural integrity (5× and 20×; scale bars: 500 μm and 100 μm). (**D**) Recellularization with MDA-MB-231 and MCF7 cell lines shows distinct growth patterns: MDA-MB-231 cells display fibroblast-like and endothelial-like features (blue and red arrows), while MCF7 cells distribute along the surface and interior of the scaffold, consistent with their luminal phenotype. Ki67 staining confirms active proliferation, with heterogeneous expression indicating both proliferative and quiescent cells (scale bars: 500 μm and 100 μm). Reproduced from [71], an open-access article distributed under the terms of the Creative Commons Attribution License (CC BY).

One of the most attractive features of dECM is the possibility to generate patient-specific scaffolds, allowing individualized tumor models and the study of patient-derived protein and genetic signatures. In their study, Landberg et al. [71] used scaffolds obtained through decellularization of breast cancer samples from various patients, which were then re-cellularized by culturing MCF-7 and MDA MB 231 breast cancer cell lines for at least 21 days (Figure 5). A heterogeneous cell differentiation was observed, with cells showing a phenotype more similar to fibroblasts or epithelial cells, thus mimicking the formation of vascular-like structures. Moreover, the analysis of gene expression revealed an important correlation between the scaffold’s protein composition and the expression of biomarkers associated with EMT. An interesting finding in the mapping of scaffold proteins associated with tumor proliferation was the tumor-suppressive nature of the periostin protein, associated with reduced tumor proliferation. A detailed, patient-specific mapping of the proteins involved in tumor onset and proliferation could serve as a useful tool in determining prognosis and potential therapies for the patient. Similarly, Romero-López et al. [72] developed an alternative scaffold system based on dECM derived from human colon tissue—both healthy and tumor metastatic samples. The decellularized matrices were processed into hydrogels that preserved the biochemical complexity and stiffness typical of their tissue of origin. Proteomic and rheological analyses revealed that tumor-derived ECM displayed a distinct matrix composition and higher stiffness than normal ECM, features known to influence cell behavior. When used as scaffolds in 3D cultures, tumor-derived ECM supported enhanced angiogenesis, with endothelial-fibroblast co-cultures forming more extensive vascular networks than in normal ECM gels. Moreover, colon cancer cells (SW620) cultured in tumor-derived ECM hydrogels exhibited accelerated growth, along with increased glycolytic metabolism, as shown by fluorescence lifetime imaging and the upregulation of metabolic genes (GLUT1, PDK1, and HXK1). These findings were confirmed in vivo, where tumor-derived ECM implants led to more vascularized and rapidly growing tumors compared to normal ECM. Overall, this study demonstrates the utility of patient-derived dECM hydrogels as physiologically relevant scaffolds that recapitulate native tumor microenvironmental conditions—offering a powerful alternative to synthetic, natural or hybrid matrices for cancer modeling and therapeutic testing.

Recent progress has been made in the development of photocrosslinkable dECM-based hydrogels for tumor and liver disease modeling. Tabatabaei Rezaei et al. [73] introduced a hybrid bioink composed of liver dECM methacrylate (LdMA) blended with GelMA, enabling rapid and tunable crosslinking under visible light irradiation. This approach overcame the limitations of traditional thermally crosslinked dECM hydrogels, which often suffer from poor mechanical stability and lack of reproducibility. The GelMA-LdMA scaffolds supported long-term culture of hepatocellular carcinoma (HepG2) cells, maintaining high viability and enhancing liver-specific functions such as albumin secretion and CYP1A2 expression. Importantly, the developed hydrogels demonstrated controlled stiffness and degradation properties, which are relevant for modeling both healthy and pathological liver microenvironments, as well as for evaluating hepatotoxic drug responses. These findings highlight how functionalized dECM bioinks may broaden the applicability of decellularized scaffolds beyond static culture, offering customizable and physiologically relevant platforms for cancer progression and drug screening studies.

Another important research direction involves modulating dECM stiffness through crosslinking strategies. In the study by Lv et al. [74], dECM was used to obtain scaffolds with varying degrees of crosslinking, by inhibiting or stimulating the expression of lysyl oxidase (LOX) through lentivirus. LOX is an enzyme involved in the enzymatic crosslinking of collagen and is generally expressed in higher quantities in tumor matrices, contributing to the stiffening of the tumor substrate. A higher degree of stiffness could be compatible with increased resistance of the cells to anticancer drugs. Furthermore, it was observed that the stiffness of certain scaffolds gradually changed with the growth of the tumor itself. Likewise, in the study by Lü et al. [75], dECM reconstituted via photo-crosslinking was used as a scaffold for the culture of various tumor cell lines: MCF-7 (from breast carcinoma), HepG2 (from liver carcinoma), and A549 (from lung carcinoma). The use of photo-crosslinking significantly increased the stiffness of the substrate without altering its chemical structure, showing greater cell density, increased secretion of interleukin-8 and VEGF, a greater tendency to migration—properties typical of EMT—and increased resistance to oncological therapies. The injection of cells cultured on photo-crosslinked dECM-based scaffolds into murine models showed a good degree of proliferation in vivo.

Thus, dECM presents promising prospects, especially in the development of patient-specific therapies and the study of specific protein and genetic components of the TME that can determine tumor prognosis. However, it still presents several application limitations, particularly in the standardization of decellularization and reconstitution procedures. The difficulty in properly controlling the mechanical properties of the matrix is undoubtedly a limitation in the creation of scaffolds with adequate structural stiffness. Additionally, it is important to consider the dynamic nature of the TME and its ability to induce and undergo structural changes during culture [70]. Technical improvements in crosslinking procedures and further studies on the chemical characterization of dECM are therefore certainly necessary to refine this promising strategy.

Importantly, the use of allogeneic or xenogeneic dECM raises additional concerns. Although decellularization reduces immunogenicity, residual species-specific antigens, damage-associated molecular patterns, or incomplete removal of cellular debris may activate immune cells once scaffolds are repopulated with tumor or immune cells [76,77,78]. Such activation can lead to non-physiological inflammatory responses, skewing the behavior of co-cultured immune populations and complicating TME modeling. Indeed, studies in regenerative medicine have shown that xenogeneic dECM can trigger macrophage polarization and T-cell activation [79,80,81]. Furthermore, crosslinking strategies such as genipin treatment have been shown to mitigate such responses by reducing antigenicity and promoting anti-inflammatory macrophage phenotypes [82].

In the oncology field, this issue is particularly relevant: Inappropriate immune activation may distort the interplay between cancer and immune cells, a central feature of the TME. For this reason, the use of human-derived or autologous dECM is increasingly advocated, despite higher costs and limited availability [83,84].

Overall, dECM scaffolds hold great promise as physiologically relevant tumor models, especially in the context of personalized oncology. However, progress toward standardization, control of mechanical properties, and mitigation of immunogenic response, particularly when using xenogeneic or allogeneic sources, will be essential to fully unlock their potential in TME research and therapeutic testing.

The main features of natural, synthetic, hybrid, and decellularized ECM-based scaffolds, along with their respective strengths, weaknesses, and representative applications in 3D tumor modeling, are summarized in Table 1.

## 5. Concluding Remarks and Future Directions

Cancer continues to represent one of the main health challenges worldwide and, despite significant advances in diagnosis and therapy, remains a leading cause of death. Reliable preclinical models are therefore essential to improve our understanding of tumor biology and to develop new therapeutic strategies. However, traditional 2D cell cultures and animal models, although widely used, fail to capture the complexity of human tumors and raise ethical and scientific concerns. In this scenario, 3D scaffold-based in vitro tumor models emerge as a promising alternative, able to replicate the TME more faithfully and to provide results with higher predictive value.

The distinctive strength of these models lies in the scaffold itself, which serves as a biomimetic and tunable support capable of reproducing the biochemical and biomechanical features of tumor tissues. By adjusting parameters such as stiffness, porosity, and composition and by incorporating TME components (fibroblasts, endothelial or immune cells) and microfluidic systems, 3D models can reproduce crucial aspects of in vivo tumor behavior—including heterogeneity, vascularization, and metastatic potential. This versatility makes them valuable not only for elucidating mechanisms of tumor progression but also for identifying new therapeutic targets and testing anticancer agents under more physiologically relevant conditions.

Different categories of biomaterials—natural, synthetic, and dECM—offer complementary advantages and drawbacks. Natural polymers confer intrinsic bioactivity and cell adhesion cues but are subject to batch-to-batch variability and unpredictable mechanical properties. Synthetic polymers, by contrast, allow tighter control over stiffness, porosity, and degradation kinetics but are often bioinert and require functionalization. dECM represents a particularly promising direction, as it can capture patient-specific biochemical cues, opening the way to personalized tumor models and individualized drug screening. This wide variety of substrates and biomaterials available, however, also complicates reproducibility and standardization—two prerequisites for turning these models into a new gold standard for oncology research.

Another major challenge is faithfully reproducing tumor heterogeneity and the dynamic interactions between cancer cells and their microenvironment. Tumor tissues are intrinsically heterogeneous, and this variability contributes to differences in therapy response and drug resistance. Co-culture systems incorporating fibroblasts, endothelial cells, adipocytes, and immune components have begun to address this issue, improving the realism of in vitro models. For example, co-cultures with cancer-associated fibroblasts (CAFs) have been shown to increase collagen deposition and matrix stiffness, thereby enhancing the resemblance to in vivo tumor stroma. Nevertheless, standardized methods for incorporating and maintaining these cell types are still lacking, and a more systematic evaluation of their impact on tumor behavior remains necessary.

Equally important is the vascular component. Vessels are not only nutrient conduits but also major players in tumor progression and metastasis formation. Cancer-on-a-chip platforms and microfluidic devices allow the integration of endothelialized channels to simulate blood flow and shear stress, offering a better representation of the tumor vasculature. Yet, paradoxically, oxygen supply is often overlooked in these models because the culture medium itself already provides oxygenation, obscuring one of the drivers of VEGF expression and abnormal angiogenesis—the formation of hypoxic cores. Future models should address this gap by mimicking oxygen gradients and vascular trophic roles more realistically.

A further layer of complexity concerns the dynamic remodeling of scaffolds by tumor cells. During culture, cancer cells secrete their own ECM, which may modify or even replace the original scaffold, potentially altering its mechanical and biochemical properties. Understanding this bidirectional interaction is crucial to clarify not only how the scaffold influences tumor evolution but also how tumor cells reshape their environment. Harnessing this process could yield even more realistic substrates and reveal new aspects of tumor–stroma crosstalk.

Overall, 3D scaffold-based tumor models are not merely a technical innovation but a paradigm shift in preclinical cancer research. They enable the study of tumor evolution, heterogeneity, and therapy response under conditions closer to the human context. To fully exploit their potential, future research should

- Define essential scaffold design parameters for different tumor types, including mechanical properties, biochemical cues, and relevant cell populations;
- Establish standardized fabrication and validation protocols to ensure reproducibility across laboratories, including clear metrics for biocompatibility, cell viability, and functional readouts;
- Incorporate dynamic and multicellular TME components, such as immune and stromal cells, vascular elements, and controlled oxygen gradients, to better model tumor complexity;
- Explore patient-specific and personalized applications, leveraging dECM and bioprinting to tailor models to individual patients for therapy testing and biomarker discovery.

By addressing these challenges, 3D scaffold-based models could bridge the gap between conventional in vitro/in vivo systems and clinical outcomes, reducing reliance on animal models, accelerating drug development, and supporting the emergence of personalized oncology.

In conclusion, the progress reviewed here shows that 3D scaffold-based tumor models represent a reliable and cutting-edge tool to study tumor evolution and to screen new anticancer therapies. Their ability to replicate key aspects of the tumor microenvironment—including mechanical properties, hypoxia, and multicellular interactions—makes them uniquely positioned to improve the predictive power of preclinical studies. While standardization and further technical refinements remain necessary, these models have the potential to become a cornerstone of future oncological research, offering not only more accurate insights into cancer biology but also a more ethical and patient-relevant platform for therapeutic innovation.

## Figures and Tables

**Table 1:** Comparison of scaffold types used in in vitro tumor models.

Scaffold Type	Biomaterials	Advantages	Limitations	Representative Applications
Natural	Collagen, Gelatin, Silk fibroin, Amyloid fibrils, Matrigel^TM^, Chitosan, Alginate, and Hyaluronic acid	High biocompatibility and bioactivity; inherent cell adhesion sites	Batch-to-batch variability; weak mechanical properties; short culture duration	Glioma [37], breast cancer [38,48,53], ovarian cancer [39,44], gastric carcinoma [40], esophageal carcinoma [41], pancreatic cancer [42,45,55], colon carcinoma [45], osteosarcoma [46], lung cancer [47,48,52], hepatocellular carcinoma [48,54], cervical carcinoma [48], and glioblastoma [50,51]
Synthetic	PLA, PLGA, and PCL	Tunable mechanical strength and degradation rates; reproducible	Low intrinsic bioactivity; required surface modification	Breast cancer [57,58,60,61] and osteosarcoma [59]
Hybrid	Combinations (e.g., Collagen + PLA, GelMA + PEGDA, Collagen + hydroxyapatite)	Balanced mechanical stability and bioactivity; customizable	Complex fabrication; compatibility challenges	Peritoneal cancer [62], prostate cancer [63], breast cancer [64,65,67], glioblastoma [66], osteosarcoma [68], and colorectal cancer [69]
dECM	Tissue-derived ECM from patients or animals	Closest mimicry of native tumor ECM; enabled personalized models	Standardization and mechanical tuning remain difficult.	Breast cancer [71,75], colon carcinoma [72], liver carcinoma [73,75], and lung carcinoma [75]

## Data Availability

No new data were created or analyzed in this study. Data sharing does not apply to this article.
